# Lattice Dynamics of Quinacridone Polymorphs: A Combined Raman and Computational Approach

**DOI:** 10.1021/acs.cgd.3c00634

**Published:** 2023-07-31

**Authors:** Andrea Giunchi, Lorenzo Pandolfi, Raffaele G. Della Valle, Tommaso Salzillo, Elisabetta Venuti, Alberto Girlando

**Affiliations:** † Dipartimento di Chimica Industriale “Toso Montanari”, Università di Bologna, Viale del Risorgimento, 4, 40136 Bologna, Italy; ‡ Molecular Materials Group, Strada Fontanini 68, 43124 Parma, Italy

## Abstract

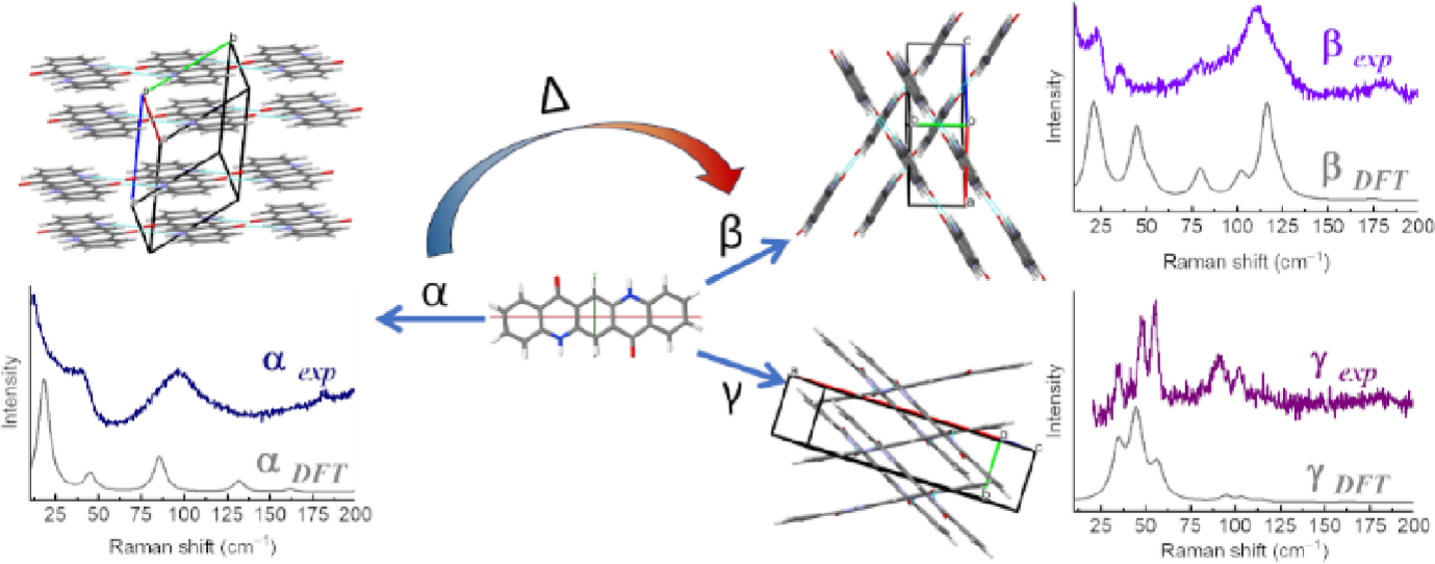

Polarized low-frequency Raman microscopy and a posteriori dispersion-corrected density functional simulations are combined to investigate the lattice vibrations of the α^I^, β, and γ polymorphs of the model organic semiconductor quinacridone, which are known to display different optical and electronic properties. The comparison between experiments and calculations allows for unambiguous mode assignment and identification of the scattering crystal faces. Conversely, the agreement between simulations and experiments validates the adopted computational methods, which correctly describe the intermolecular interaction of the molecular material. The acquired knowledge of quinacridone lattice dynamics is used to describe the α^I^ to β thermal transition and, most consequentially, to reliably characterize the electron–lattice phonon coupling strength of the three polymorphs, obtaining hints about the electrical transport mechanism of the material.

## Abstract 2

Lattice vibrations of the α^I^, β, and γ polymorphs of the model organic semiconductor quinacridone are investigated combining polarized low-frequency micro-Raman and dispersion-corrected density functional simulations. The study of the lattice dynamics of the system allowed for the identification of the α^I^ to β thermal transition and the characterization of the electron−lattice phonon coupling strength of the three polymorphs.

## 1 Introduction

Transport characteristics of crystalline organic crystals are influenced and shaped by their vibrational properties.^1^ Their anisotropic thermal conductivity is mostly contributed by phonons; also, theoretical treatments point to electron–phonon coupling as one of the mechanisms responsible for energy dissipation of excitons and charge carriers in crystalline organic semiconductors.^1−3^

In principle, the most intriguing property of organic materials is the chemical tunability of their structural and electronic properties, as they can be tailored by synthesis, while in turn, crystal engineering may assist establishing molecular arrangements, which enhance transport parameters via intermolecular interactions. In practice, however, properties cannot be easily adjusted purely by design,^1^ and this makes the effects of chemical and structural modifications on its dynamics (i.e., its vibrational characteristics) badly predictable.

The above considerations suggest that, among the investigations needed to characterize a molecular functional material, the computational assessment of its vibrational properties should be included, with special emphasis on the low-energy side, where lattice phonons, that is, the collective motions, which originate from the crystal periodical arrangement and mostly depend on the intermolecular interactions, can be found.^4−7^

From an experimental point of view, inelastic neutron scattering allows for the investigation of vibrational modes over the full extent of the first Brillouin zone, but it is not a readily accessible technique and, apart from the determination of the density of states (DOS), data analysis is not a simple task. Raman spectroscopy is an easily accessible tool, and although it only probes the Γ-point of the first Brillouin zone, this limitation does not matter for modes with little or no dispersion. Above all, being the method of choice for the detection of the low-energy portion of the vibrational spectrum, it constitutes a direct validation of computations, which include the description of the intermolecular interactions responsible for the transport characteristics of the molecular material.

**Scheme 1:**
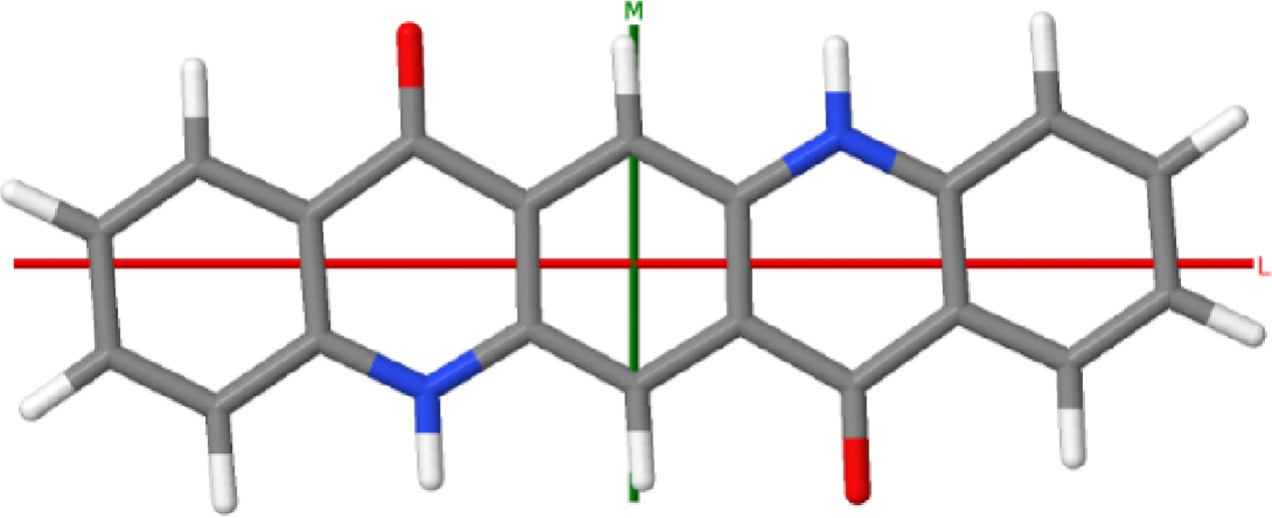
Molecular Structure of Quinacridone Inertia axes L and M for the molecular geometry in the crystal are also drawn; the axis N is normal to the molecular plane.

In this work, the combination of Raman experiments and a posteriori vdW-corrected DFT simulations^8−10^ is applied to the characterization of the low-frequency lattice dynamics of the organic semiconductor quinacridone^11^ (5,12-dihydroquinolino[2,3-*b*]acridine-7,14-dione, QA, Scheme 1). Quinacridone is an artificial dye that has received a renewed interest as a model system^12,13^ for charge transport in organics because of its many crystal forms,^14^ with the polymorphism originating from the simultaneous occurrence of dispersion, π–π stacking, and H-bonding interactions. In fact, because H bonding is important in the packing, it cannot be considered a simple vdW system. As such, it represents a critical benchmark of the computational approach.

In previous works,^15,16^ we gave a detailed account of how the combination of different spectroscopic techniques (Raman, infrared, and luminescence), also applicable to a working device, can be used to identify the different quinacridone crystal structures. Here, we show that the DFT simulations of the vibrational properties at the Γ point reproduce the low-frequency Raman spectra of all polymorphs with high fidelity. The detailed information on the spectral features of the polymorphs is put to use in the description of the transition from α^I^ to β forms. Most importantly, the reliable description of the lattice phonon eigenvectors of all polymorphs given by the DFT simulations allows for a safe estimate of the corresponding Peierls coupling constants, with hints on their expected different charge mobility behavior.

## 2 Experimental and Computational Methods

Raman spectra of powder samples and/or single crystals of α^I^-QA, β-QA, and γ-QA polymorphs were obtained as reported in ref (15). Microcrystalline samples of α^I^-QA suitable for the measurements were also obtained by thermal cleavage of the crystal form II of the chemical precursor ^*t*^Boc-QA,^17^ following the procedure of ref (16). Specifically, spectra in the lattice phonon region (10–200 cm^–1^) were collected with a Horiba Jobin Yvon T64000 spectrometer equipped with three monochromators in double subtractive configuration and a Peltier cooled charge-coupled device (CCD, Horiba Syncerity) as a detector. The spectrometer was coupled to an Olympus BX40 confocal microscope equipped with 100×, 50×, 20×, and 10× objectives, with a lateral resolution of ≈1 μm with the 100× objective. Such an objective was the one employed for all measurements, while those with lower magnification were used to aid the selection of the crystal area from where the scattered light was to be collected. Spectra were collected with the excitation wavelength of the 647.1 nm line of a Kr^+^ gas laser (Coherent Innova 90C), at a resolution of ≈1 cm^–1^ over the spectral window 5–250 cm^–1^, and were calibrated by the detection of the 650.7 nm line of a neon lamp. Despite the known thermal and photo stability of QA,^11^ the laser power was kept below 1 mW with neutral density filters to avoid possible sample damage due to absorption processes at this wavelength. A half-wave plate was used to rotate the polarization of the incident light, while a wire grid polarizer selected the polarization of the scattering. Normally, summing over 5 measuring cycles of 120 s integration time each was sufficient to acquire spectra with a satisfactory S/N ratio. All measurements were carried out at room temperature, with the exception of the thermal cleavage of QA chemical precursor and α^I^-QA to β-QA phase transition, with the temperature control provided by a Linkam LST420 hot stage. Previously reported X-ray indexing on crystal faces,^15^ morphology, and extinction directions allowed us to orient the crystal specimens for measurements in polarized light. Literature crystal structure parameters with the figures of the cell unit arrangements^14^ are reported in the Supporting Information.

DFT simulations on all the crystal forms were performed using the code VASP (Vienna ab initio simulation package).^18−21^ The Perdew–Burke–Ernzerhof (PBE) exchange correlation functional^22^ was employed together with projected-augmented wave (PAW) pseudopotentials.^23,24^ The effects of the dispersive interactions were included with the D3-BJ pair-wise correction by Grimme et al.^25^ For all QA polymorphs, a plane wave cutoff of 800 eV proved to be adequate to achieve energy convergency in combination with Monkhorst–Pack k-point samplings of 3 × 2 × 1, 3 × 4 × 1, and 1 × 5 × 1 for forms α^I^, β, and γ. Checks were made by increasing the cutoff from 800 to 1200 eV for which energy variations around 0.05 meV/atom were obtained. The calculations were performed at the experimentally determined unit cell parameters of each structure, using the data at 293 K given in ref (14), and relaxing the atomic coordinates by means of the GADGET package^26^ until the residual forces fell below 1 meV/Å. The obtained structures correspond to a constrained minimum of the potential energy surface of the crystal, with no vibrational contribution. Following the protocol described in ref (10), for all the minimized structures, the Γ point vibrational modes were computed at the corresponding fixed unit cell parameters to optimize the agreement with the experiments, with the force constants obtained by the PHONOPY software^27^ in combination with VASP. To this aim, the software pipeline developed by us for previous works is used.^8,28^ In detail, the crystal space group, as detected by the SPGLIB library,^29^ is used to build the symmetry coordinates for all the irreducible representations of the group. The matrix of the force constants produced by PHONOPY is thus reduced to a block-diagonal form, with a single block for each representation component. Each block is then separately diagonalized, yielding vibrational eigenvalues (i.e., frequencies) and eigenvectors for that component. To assist with the assignment of the vibrational modes, beside the symmetry, at this stage, we also evaluate the projection of the eigenvectors onto rigid translations and rotations of the molecules, computed at the equilibrium centers of mass and inertia axes (i.e., the Eckart frame^30^). Polarizability tensors for each crystal mode were obtained by using the Python program vasp_raman.py,^31^ which uses the VASP code as backend. Raman intensities were finally adjusted by considering excitation wavelength and temperature dependence.^32^ The details of the procedure followed to calculate the unpolarized (powders samples) and polarized Raman spectra are reported in the Supporting Information.

The Peierls or electron–lattice phonons coupling constants were calculated following the usual protocol.^1,33^ The intermolecular hopping or transfer integrals *t* between the frontier orbitals of QA dimers were computed as one-electron HOMO–HOMO (LUMO–LUMO) coupling following the dimer projection approach^34^ using the ORCA v5.0 package^35^ with the PBE0 functional and def2-SVP basis set. We verified that the use of a more extended basis like def-TZVP does not alter significantly the obtained numerical values of transfer integrals and Peierls coupling constants. The latter were obtained by a numerical derivative of the transfer integral with respect to the lattice phonon eigenvectors. Only the transfer integrals and coupling constants relevant to the HOMO (valence band) are reported here.

## 3 Results and Discussion

We have focused on three QA polymorphs, namely α^I^, β, and γ.^14,36^ The α^I^ form is a triclinic P1̅ structure, which shares with monoclinic P2_1_/c β form the H-bonding motif, where each molecule is connected to two neighbors.^36^ This generates packings in which chains of molecules may get differently arranged, producing a variety of structures. The form γ, also monoclinic P2_1_/c, is instead characterized by an altogether distinct H-bonding motif, with each molecule connected to four neighbors in a crisscross pattern.^36^ Number and temperature relative stability of quinacridone polymorphs have been the subject of debate. Their number was clarified by Paulus et al.,^14^ with no conclusive indication of their thermodynamic temperature range of existence. It is generally accepted that the α^I^ form is unstable at all temperatures; in fact, despite being often obtained in industrial synthesis,^14,36^ it transforms into either β or γ by thermal treatment, and no reverse transition is known. The γ phase is about 3% less dense than the β, but according to experimental observations is the most stable, at least at high temperatures. In a posteriori dispersion-corrected energy calculations^13^ carried at the experimental volumes, which did not include the vibrational contribution, the α^I^ form was found less stable than β and γ by ∼2.0 Kcal/mole, suggesting that is not the stable phase at low *T*. The β form was instead calculated less stable than γ by ∼0.1 Kcal/mole.

Measurements and simulations of the QA polymorph vibrational properties have been performed in the wavenumber range up to 200 cm^–1^. This corresponds to the spectral window of thermal vibrations and roughly represents the boundary between modes that can be classified in organic solids as inter- or intramolecular in character. Although very useful, the idea of a complete separation between the degrees of freedom is somehow notional because of the presence of vibrations corresponding to torsions and internal rotations. In fact, a vibrational frequency calculation made on the QA-isolated molecule with C_2h_ molecular symmetry (see Supporting Information) shows that despite having a quite rigid skeleton, this system possesses three low-frequency out-of-plane modes of B_g_ symmetry below 200 cm^–1^ and two in-plane modes of A_g_ symmetry below 250 cm^–1^. The way that these contribute to shape the low-energy portion of the crystal vibrations, in spectral pattern and intensities, greatly depends on the polymorphic structure.

**Figure 1:**
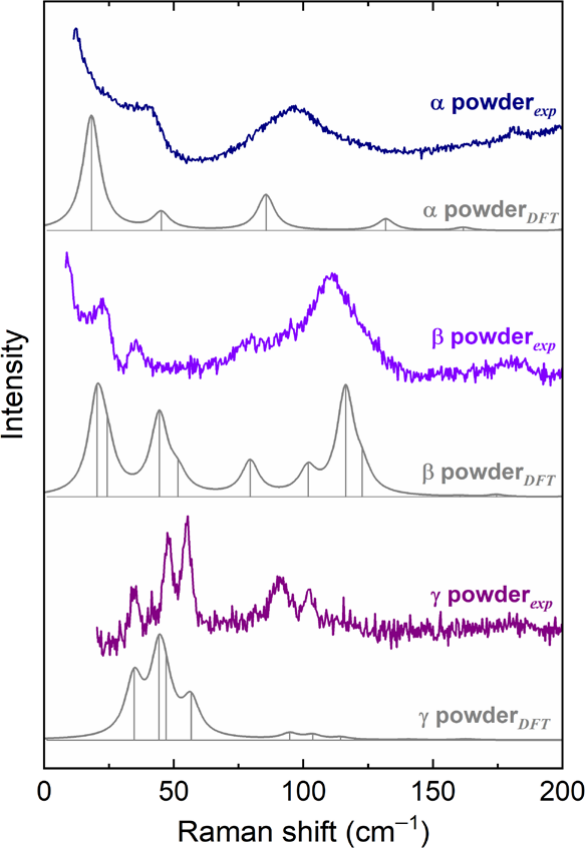
Simulated and experimental Raman spectrum of α^I^-QA, β-QA, and γ-QA in the wavenumber range of the lattice phonons up to 200 cm^–1^. The corresponding bands have been drawn as Lorentzian bands with FWHMs of 4 cm^–1^ chosen to conform to the experimental features.

The low-frequency experimental Raman spectra of the powders of α^I^,β, and γ-QA are reported in Figure 1, along with the corresponding simulated ones for powder-like samples. The spectra of the three polymorphs are remarkably different, allowing easy discrimination,^14^ but what we want to stress in the present context is the fact that not only the frequencies but also the intensity pattern is in general well-reproduced by the calculation.^10^ Below, we proceed to a detailed interpretation and spectral assignment for each polymorph.

### 3.1 α^I^-QA Polymorph

The molecular symmetry lowering in α^I^-QA with respect to the isolated species involves a relaxation of the spectroscopic selection rules, which apply in the gas phase. As the molecular inversion center is retained in the site symmetry of the triclinic P1̅ structure with one molecule per cell and at wavevector **k** = 0 (i.e., at the Γ point), the vibrational modes of gerade A_g_ symmetry are Raman active. Keeping separated inter- and intramolecular degrees of freedom, three of these modes are lattice phonons and identify with concerted rotations of the unit cell molecule around its inertia axes L, M, and N drawn in Scheme 1. The remaining modes originate from molecular vibrations of gerade symmetry of the isolated species.

Table 1 reports the values of the experimental peaks (top trace of Figure 1) compared to the vibrational eigenvalues in the same range for α^I^-QA along with the eigenvector analysis of the (squared) rotational components around the three inertia axes. Such components sum up to ≈100% (unity) for at least two of the modes and amount to 76% for a third one. The animations for these modes are given in the Supporting Information. The intense pure lattice mode calculated at 86 cm^–1^ (experimental 97 cm^–1^) corresponds to a pure libration around the long in-plane inertia axis L of the molecule (see Scheme 1). The mode at 45 cm^–1^ (experimental 41 cm^–1^) has the mixed character of a libration around the inertia axis M and a molecular torsional motion. Both the calculated out-of-plane B_g_ modes of the isolated molecule with C_2h_ symmetry lying at 65 and 134 cm^–1^ (Figure S1) lose their B character in the crystal site symmetry and can combine to give rise to the observed crystal mode. Conversely, the modes calculated at 132 and 162 cm^–1^ most closely resemble the two isolated molecule vibrations.

**Table 1:** Calculated and Experimental Wavenumbers of the Low-Energy Raman Active Modes of α^I^-QA Polymorph and Calculated Squared Rotational Components around the Three Inertia Axes L, M, and N (See Scheme 1)

exp (cm^–1^)	DFT (cm^–1^)	%rotations		
	18.2	0	1	97
41	45.2	1	75	0
97	85.6	95	2	1
125	131.8	2	17	0
181	161.7	1	3	1

The lattice mode calculated at 18 cm^–1^ corresponds to the rotation around the axis N normal to the molecular plane and displays the largest intensity despite the highest inertia moment but is undetected by the experiment. This last point, however, deserves some comments. The α^I^-QA polymorph is known to be characterized by extended disorder so that no single crystals could be obtained. This results in broad bands in the Raman spectrum of Figure 1. Moreover, the disorder also causes the onset of the so-called Rayleigh wing, which dominates the lowest portion of the experimental spectrum and prevents a clear determination of the lowest energy peak. The wing is a clear signature for disorder and has been detected, for instance, in systems described as “glassy crystals”.^37^

### 3.2 β-QA Polymorph

In the monoclinic P2_1_/c of β-QA, the *Z* = 2 molecules of the unit cell lie on inversion centers, which lowers the QA molecular symmetry in the crystal to C_i_, like for α^I^-QA. At **k** = 0, the (108) Raman active modes are equally distributed in 54 A_g_ + 54 B_g_, and six of them (3A_g_ + 3B_g_) must hold the character of lattice modes. Frequencies, symmetry, and eigenvector analysis of the β-QA modes computed up to 200 cm^–1^ are given in Table 2, along with the Raman experimental values for the crystal. The Raman spectrum of the powder sample of Figure 1 serves as a useful comparison.

**Table 2:** Calculated and Experimental Wavenumbers of the Low-Energy Raman Active Modes of β-QA Polymorph with Symmetry Assignment and Calculated Squared Rotational Components around the Three Inertia Axes L, M, and N (See Scheme 1)

exp (cm^–1^)	DFT (cm^–1^)	symmetry	%rotations		
	20.4	A_g_	0	89	11
27	24.3	B_g_	0	43	57
39	44.4	A_g_	0	10	87
	51.6	B_g_	2	40	87
78	79.5	A_g_	21	1	2
96	101.8	B_g_	40	14	5
113	116.3	A_g_	78	0	0
125	122.6	B_g_	56	3	1
	160.4	A_g_	0	0	0
	174.5	B_g_	1	0	0

The β-QA phase can be grown as single crystals from the commercial powder by the physical vapor transport (PVT) method, at a deposition temperature of about 300 °C.^15^ The crystals have been found by XRD to have *ab* as the dominant face,^15^ and this allows for an efficient polymorph discrimination also in polarized mid-IR spectroscopy, where their features differ from the γ-QA form, which instead has *bc* as the dominant face (see below). Very often, however, the samples display an irregular shape and twinning, and therefore, the Raman scattering is likely to contain the contribution of other crystallographic faces, as suggested by the large collection of spectra analyzed for this work. In fact, as each mode gathers intensity from the elements of the polarizability matrix selectively probed on a specific face, the relative intensities of the same bands can display a large dependence on the sample orientation. Specifically, in the reference framework of the monoclinic crystal, the intensities of the A_g_ modes depend on the α_*aa*_, α_*bb*_, α_*cc*_, and α_*ac*_ matrix elements, while those of the B_g_ modes depend on elements α_*ab*_ and α_*bc*_.

**Figure 2:**
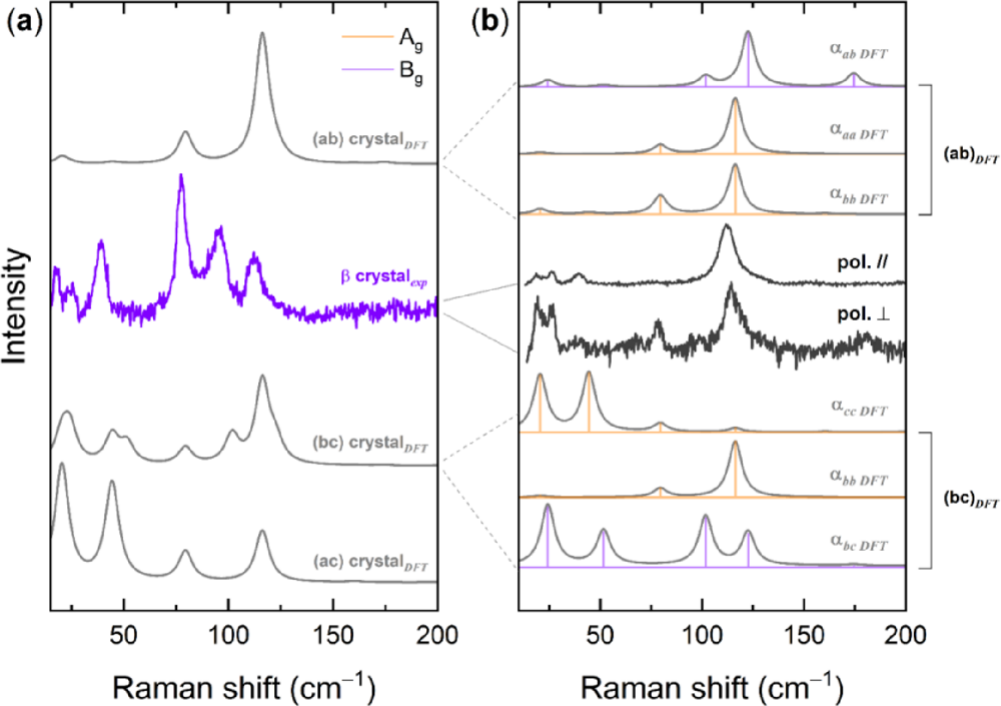
(a) Simulated DFT Raman spectra for the *ab*, *ac*, and *bc* crystallographic faces of β-QA monoclinic structure compared to experiments on a crystal. (b) Polarized simulated DFT Raman spectra of crystal faces *ac* and *bc* compared to the experiments on a crystal. Calculated intensities are given as yellow lines for the modes of A_g_ symmetry and violet lines for the modes of B_g_ symmetry. The corresponding bands have been drawn as Lorentzian bands with FWHMs of 4 cm^–1^, chosen to conform to the experimental features. For each spectrum, the selected polarizability matrix element is also indicated. Experiments are referred to as conducted with parallel or crossed polarizers.

The simulated unpolarized spectra of the main faces of the β*-*QA crystal are given in Figure 2a. The spectra correspond to an experiment in which the light is scattered from the face of a randomly oriented sample, and no polarization discrimination is applied in either excitation and detection. The DFT intensities for any given face are given by the sum of the components of eqs S4–6 as in following:
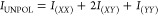


The simulations of Figure 2a show that the β-QA modes below 80 cm^–1^ should all appear as weak on face *ab*, in contrast to what experimentally observed for most specimens. However, based on the good agreement with the experimental powder spectrum of the figure, the identification of the detected faces and/or intensity components can be made using simulated spectra. This is done for one typical sample in Figure 2b, where a series of spectra in polarized light, obtained by orienting the crystal along its light extinction directions, are compared to the simulations for both faces *ab* and *bc*. Overall, the experiments appear to detect face *ab*, which agrees with the finding that this is the dominant face for β-QA. Nonetheless, some features, such as the intensities displayed at low wavenumbers, are better reproduced by the contribution of modes that gather intensity from the α_*cc*_ and α_*bc*_ matrix elements, suggesting that *ab* is not the only face observed. In fact, the spectrum of the totally symmetric A_g_ modes is rendered by the combination of *I_xx_* and *I_yy_* terms of the simulations for both faces *ab* and *bc*, demonstrating that the examined sample was not a single crystal. This also accounts for the residual polarization clearly present in some of the experimental spectra reported in the literature.^9^

Once clarified the characteristics of the experiments, the mode assignments can be made based on the polarized spectra and Table 2 data. The bands at 39, 78, and 113 cm^–1^ all belong to modes of A_g_ symmetry, whereas the bands at 26, and 115 cm^–1^ and the weak one at 125 cm^–1^ to modes of B_g_ symmetry. Two bands of A_g_ and B_g_ symmetry go undetected in the experiments, in agreement with the simulation prediction of very low intensities. Similar to what happens for the α^I^-QA phase, the analysis of β-QA eigenvectors shows that the simplified picture of uncoupled inter- and intramolecular modes does not fully apply, as the lattice mode character is distributed over more than the six expected vibrations. Pure lattice modes are the concerted vibrations about the M and N inertia axes. However, for instance, the A_g_ mode calculated at 79 cm^–1^ (experimental at 78 cm^–1^) amounts only for the 21% to the concerted rotations about the axis L of the two molecules in the unit cell and has a very close correspondence in the isolated molecule modes at 65 and 134 cm^–1^.

### 3.3 γ-QA Polymorph

As stated above, γ-QA shares with the β polymorph, the P2_1_/c monoclinic crystal symmetry, but its structure is characterized by an altogether different arrangement of the molecules in the unit cell, leading to a distinctive H-bonding pattern among all the QA crystal forms. This, in turn, results in specific spectroscopic signatures for this thermodynamically stable polymorph. With *Z* = 2 molecules per cell, the vibrational analysis for γ-QA is the same as for β-QA, with 54 A_g_ + 54 B_g_ Raman active modes, six of which are lattice phonons (3A_g_ + 3B_g_). Frequencies, symmetry, and eigenvector analysis of the modes computed up to 200 cm^–1^ are given in Table 3, along with the Raman experimental values of the band peaks.

**Table 3:** Experimental and Calculated Wavenumbers of the Low-Energy Raman Active Modes of γ-QA Polymorph with Symmetry Assignment and Calculated Squared Rotational Components around the Three Inertia Axes L, M, and N (See Scheme 1)

exp (cm^–1^)	DFT (cm^–1^)	symmetry	%rotations		
35	34.7	A_g_	12	1	87
43	44.3	A_g_	16	84	0
48	47.0	B_g_	33	25	36
55	56.6	A_g_	72	14	13
55	56.7	B_g_	9	70	18
91	94.7	B_g_	37	0	13
101	103.6	A_g_	0	0	0
	114.4	B_g_	19	2	31
	140.7	A_g_	0	0	0
	162.7	B_g_	0	2	0

**Figure 3:**
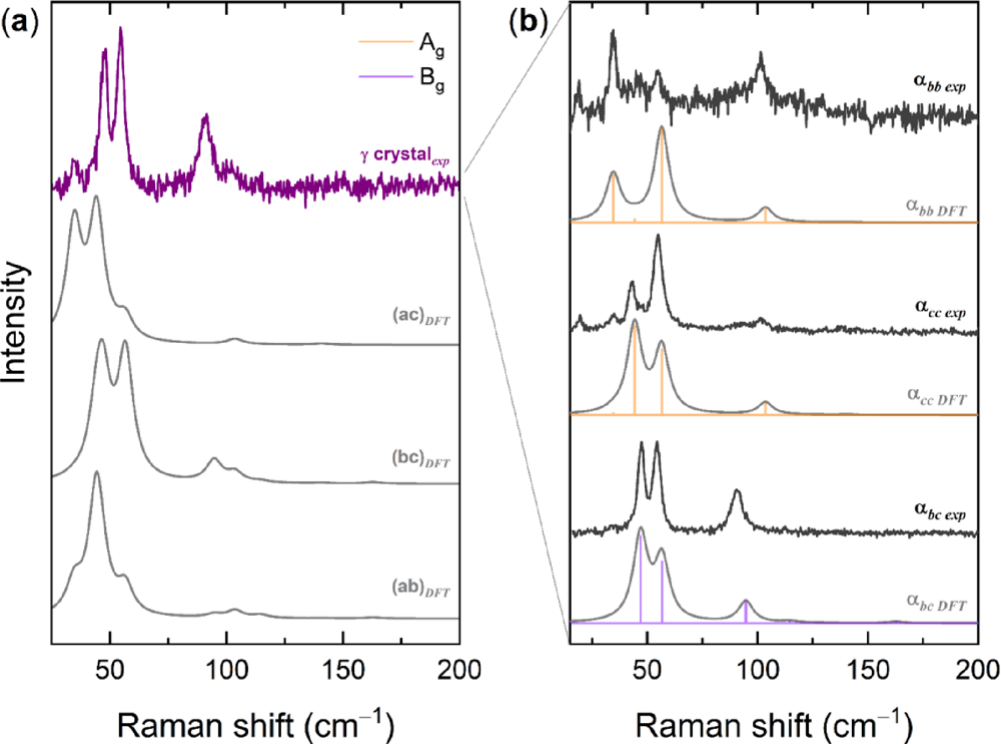
(a) Simulated DFT Raman spectra for the *ab*, *ac*, and *bc* crystallographic faces of γ-QA monoclinic structure compared to experiments on a crystal. (b) Polarized simulated DFT Raman spectra of crystal face *bc* compared to the corresponding experiments performed on a crystal. Calculated intensities are given as yellow lines for the modes of A_g_ symmetry and violet lines for the modes of B_g_ symmetry. The corresponding bands have been drawn as Lorentzian bands with FWHMs of 4 cm^–1^ chosen to conform to the experimental features. For each simulated and experimental spectrum, the selected polarizability matrix element is also indicated.

The simulated Raman spectra for all main crystallographic faces of γ-QA are reported in Figure 3a, along with the experiment on the crystal. The comparison between calculated and experimental polarized spectra (Figure 3b), recorded by orienting the crystal along the extinction direction, that is, parallel to the crystal axis *b*, confirms that the detected face is *bc*, in agreement with the XRD datum.

Five of the low wavenumber modes of γ-QA lying at energies below 60 cm^–1^ are found by the simulations to correspond to pure lattice vibrations. The three A_g_ bands computed at 35, 44, and 57 cm^–1^ (experiments at 35, 43, and 55 cm^–1^) correspond to concerted librations with major components around axes N, M, and L, respectively, in agreement with the trend of the corresponding inertia momenta. The phonon lattice B_g_ mode of lowest energy (calculated 47 cm^–1^, experimental 48 cm^–1^) has a mixed L, M, and N character, while the second one has a major component around the axis M (calculated 57 cm^–1^, experimental 55 cm^–1^). The remaining lattice phonon character of B_g_ symmetry is almost equally distributed among the modes calculated at 95 and 114 cm^–1^, with the summed squared rotational components that amounts to ≈50%50%. However, only a band at 91 cm^–1^ is experimentally detected, in agreement with the very low intensity predicted by the simulations for the one at higher energy.

### 3.4 α^I^-QA to β-QA Phase Transition

The detailed knowledge of the spectral properties of QA in the lattice phonon region can be applied to the study of the thermal α^I^*-*QA to β-QA phase transition. As mentioned above, the α^I^-QA polymorph can be obtained in the synthesis processes and gets transformed either into β-QA or γ-QA by various post-treatments; in fact, depending on the reaction conditions, all phases or most often mixtures of them can be synthesized.^14,36,38,39^ A safe way of obtaining pure α^I^-QA in the thin film phases is via the thermal cleavage of the P2_1_/c polymorph (form II) of chemical precursor ^*t*^Boc-QA. This selectively triggers the formation on silicon oxide of the semiconductor α^I^ metastable structure, whereas upon heating the most common ^*t*^Boc-QA form I, β-QA is obtained.^16^ This finding exemplifies how exerting control over the crystalline phase of a given compound may require knowledge of the chemical and thermal history of the sample.

**Figure 4:**
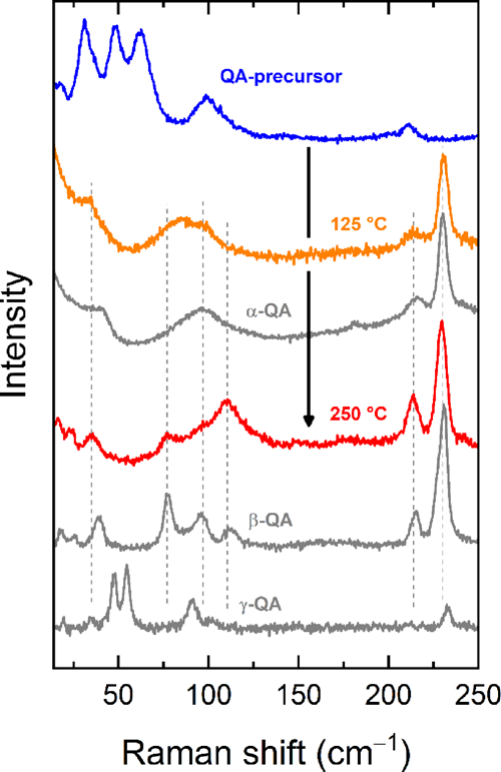
From top to bottom: the synthesis of the α^I^-QA polymorph by thermal cleavage of a crystal form II of the chemical precursor ^*t*^Boc-QA is detected by lattice phonon Raman spectra (orange trace); the obtainment of the β-QA on increasing temperature is also detected by the Raman measurements.

The crystal-to-crystal transformation that leads from ^*t*^Boc-QA form II to α^I^-QA is not limited to the thin film phases of the two compounds. In fact, as shown in Figure 4, also by heating at 125 °C the needle-like single crystals of form II, the α^I^-QA phase is recovered, with a lattice phonon spectrum which retains the characteristics displayed in Figure 1, and a phase assignment supported by the simulations for this polymorph. This suggests that rather than by the orientation of the precursor molecules with respect to the substrate, the formation of the α^I^-QA polymorph is determined by its bulk packing characteristics in form II.

On increasing the temperature up to 250 °C, the change of the lattice phonon pattern intercepts a transformation, which neither causes an evident change of the microcrystal color nor sample disruption. The identification of the product of the phase transition as being β-QA is made possible by the detection of the bands at 39, 78, 96, and 113 cm^–1^. The comparison with the simulations of both Figures 1 and 2 allows for a reliable peak assignment of this polymorphs and shows that it obtained as the crystalline powder.

### 3.5 Peierls Coupling Constants

Understanding and modeling the charge transport mechanism in van der Waals molecular organic semiconductors is a complex endeavor, as their mobility generally is in between the regime properly described in terms of incoherent hopping and the conventional band transport.^1−4^ Recently developed theories have pointed out to the role of low-frequency, intermolecular phonons and of their coupling to the electronic system (Peierls coupling). Quinacridone is not a simple van der Waals solid, as H bonding plays an important role in the packing. At the same time, its operational mobility has been shown to depend strongly on the involved polymorph and sample preparation, ranging from ∼10^–4^ cm^2^ V^–1^ s^–1^ for α^I^-QA and β-QA^16^ to values comparable to pentacene (∼0.1 cm^2^ V^–1^ s^–1^) for slowly evaporated layers of β-QA^40^ and γ-QA.^11^ Despite these interesting mobility values, very little theoretical modeling has been made on the QA electronic structure and the relevant parameters.^12,13^ In particular, to the best of our knowledge, the evaluation of the Peierls coupling strength has not been reported so far. Therefore, having determined reliable eigenvectors for the lattice phonons of the three QA polymorphs, we report in the following an estimate of the transfer integrals and of the corresponding Peierls coupling constants.

**Figure 5:**
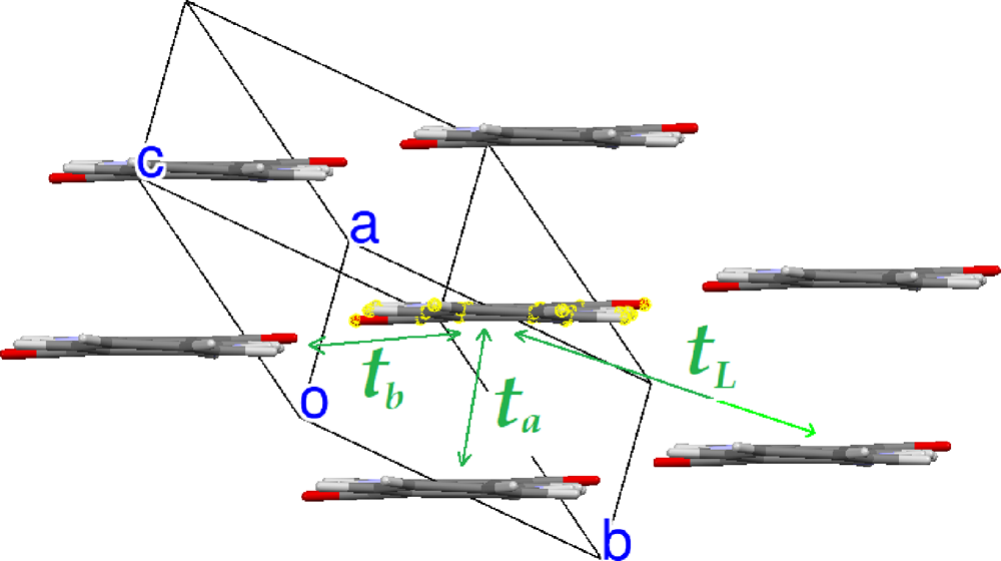
Main hopping integrals of α^I^-QA. Calculated values: *t_a_* = 15.5 meV, *t_b_* = 10.2 meV, and *t_L_* = 12.6 meV.

**Figure 6:**
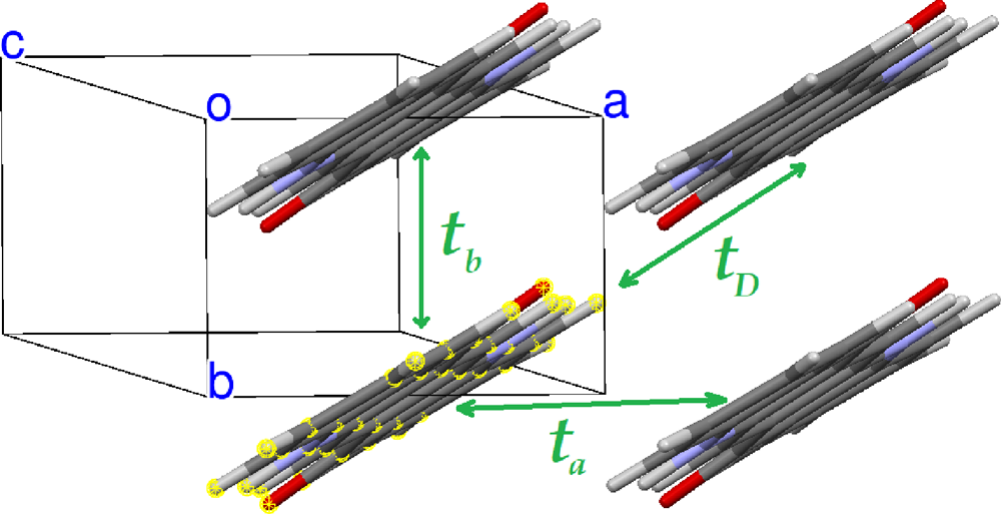
Main hopping integrals of β-QA. Calculated values: *t_b_* = 47.5 meV, *t_a_* = 8.9 meV, and *t_D_* = 14.6 meV.

**Figure 7:**
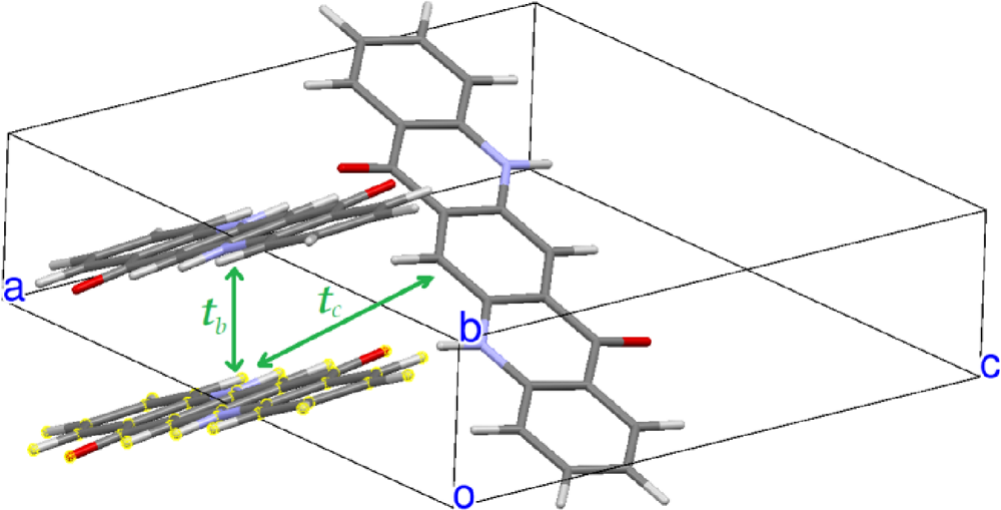
Main hopping integrals of γ-QA. Calculated values: *t_b_* = 11.7 meV and *t_c_* = 8.7 meV.

We first present all the nearest-neighbor hopping integrals *t_rs_* = ⟨ϕ*_r_* | ***H*** | ϕ*_s_*⟩ between QA pairs in the three polymorphs (***H*** is the one-particle Hamiltonian, and ϕ_*r*_ and ϕ_*s*_ are the relevant molecular HOMOs at sites *r* and *s*). The labelling of the main hopping integrals and their calculated values for the equilibrium structures are reported in Figures 5–7.

The transfer integrals relevant to pairs of QA molecules roughly facing head-to-head, that is, along the L axis of Scheme 1, are practically zero in all the three polymorphs. On the other hand, the QA transfer integrals are on the whole rather small compared, for instance, with those of pentacene. The largest one is found along the π–π stacking direction of β-QA, *t_b_* in Figure 6. This finding is in agreement with ref (13), whereas those of the γ phase appear to be appreciably smaller. Considering the relative values of the transfer integrals for each polymorph, we would qualitatively expect 2D transport properties for the α^I^-QA and γ-QA polymorphs, whereas those of the β phase appear to be more remarkably 1D along the *b* crystallographic axis.

Now, we turn attention to the calculation of the Peierls coupling constants, defined as:^33^
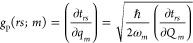
where *q_m_* and *Q_m_* are the dimensionless and dimensional (spectroscopic) normal coordinates of the (zone center) optical mode *m* of frequency ω_*m*_, respectively. The strength of the Peierls coupling is expressed by the lattice distortion energy ϵ_d_:
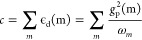


Another aspect of the lattice distortion energy is that, in the room temperature limit (as in the present case), it is connected to the variance σ^2^ of the transfer integrals due to thermal fluctuations:^41^ σ^2^ = 2ϵ_d_*k*_B_*T*. In qualitative terms, ϵ_d_ is a measure of the dynamic disorder connected to the thermal lattice fluctuations.

The computed Peierls coupling constants of the three QA polymorphs are given in Tables 4–6. Since only values greater than 1.0 meV are reported, all the coupling constants relevant to *t_D_* of β-QA and all those relevant to *t_c_* of γ-QA are not present in Tables 5 and 6, being below this threshold. We also checked the coupling constants of the ungerade (infrared active) lattice phonons. In most case, they are zero by symmetry, but also when symmetry is not involved, they are below 1.0 meV.

**Table 4:** Peierls Coupling Constants of α^I^-QA^a^

exp (cm^–1^)	DFT (cm^–1^)	*g*_P_^(*a*)^ (meV)	*g*_P_^(*b*)^ (meV)	*g*_P_^(*L*)^ (*meV*)
	18.2	1.8	1.8	
41	45.2	–7.5	1.1	
97	85.6	–5.9	1.3	2.3
125	131.8	6.4	–0.7	
181	161.7	4.3		1.5

a Only *g*_P_ values greater than 1.0 meV are reported.

**Table 5:** Peierls Coupling Constants of β-QA^a^

exp (cm^–1^)	DFT (cm^–1^)	symmetry	*g*_P_^(*b*)^ (meV)	*g*_P_^(*a*)^ (meV)
	20.4	A_g_	–2.2	
27	24.3	B_g_	–1.1	
39	44.4	A_g_	–1.6	
	51.6	B_g_	–4.8	
78	79.5	A_g_	–4.6	
96	101.8	B_g_		1.5
113	116.3	A_g_	–11.4	–3.4
	122.6	B_g_	–11.5	–3.3
	160.4	A_g_	–2.2	–2.3
	174.5	B_g_	–2.6	–1.9

a Only *g*_P_ values greater than 1.0 meV are reported.

**Table 6:** Peierls Coupling Constants of γ-QA^a^

exp (cm^–1^)	DFT (cm^–1^)	symmetry	*g*_P_^(*b*)^ (meV)
35	34.7	A_g_	–2.0
43	44.3	A_g_	–4.8
48	47.0	B_g_	
55	56.6	A_g_	
55	56.7	B_g_	–3.6
91	94.7	B_g_	
101	103.6	A_g_	1.8
	114.4	B_g_	2.5
	140.7	A_g_	1.9
	162.7	B_g_	1.2

a Only *g*_P_ values greater than 1.0 meV are reported.

In general, the values of the coupling constants are below 10 meV and rather uniformly distributed. Only the two phonons of β-QA, at 116.3 and 122.6 cm^–1^, one A_g_ and one B_g_, have values around 11 meV in the π–π stacking direction (*b* axis). A scheme of the eigenvectors of the two modes is given in Figure S2.

Table 7 summarizes the values of the transfer integrals and the associated lattice distortion energy. We notice that in the case α^I^-QA, the lattice distortion energy along the π–π stacking direction is even greater that the transfer integral, meaning strong dynamic disorder in that direction, which sums up to the static disorder that hampered a proper structural analysis:^14^ no surprise that the mobility of α^I^-QA is lower than those of the other two polymorphs. It is instead surprising that the mobility of γ-QA^11^ is comparable to that of sublimed β-QA,^40^ considering that the transfer integrals are much smaller. Of course, one must keep in mind that we are comparing operational mobilities, strongly dependent on the device fabrication.^11,16,40^ But we have also seen that in the case of β-QA, apparently well-grown crystal faces will likely present directional disorder and that there are the two abovementioned “killer modes” along the stack direction, two facts that may easily hamper transport in a strongly anisotropic (quasi 1D) semiconductor. On the other hand, mobility in γ-QA might be favored by the fact that the naturally grown *bc* crystal face of the device is the one where the transport may more easily occur, also considering the virtually null lattice distortion energy along the *c* direction (Table 7).

**Table 7:** Transfer Integrals and Lattice Distortion Energies (meV) for the Three QA Polymorphs

α^I^-QA	*t_a_ =* 15.5, ϵϵ_d_^(*a*)^ = 18.2	*t_b_ =* 10.2, ϵ_d_^(*b*)^ = 2.8	*t_L_ =* 12.6, ϵ_d_^(*L*)^ = 0.8
β-QA	*t_b_ =* 47.5, ϵ_d_^(*b*)^ = 17.8	*t_D_ =* 14.6, ϵ_d_^(*D*)^ ∼ 0	*t_a_ =* 8.9, ϵ_d_^(*a*)^ = 2.1
γ-QA	*t_b_ =* 11.7, ϵ_d_^(*b*)^ = 8.0	*t_c_ =* 8.7, ϵ_d_^(*c*)^ = 0.2	

## 4 Conclusions

In this work, we have employed the DFT simulations of the low-frequency vibrational spectra of quinacridone polymorphs to improve the understanding of the spectral features displayed by the various crystal structures of this OSC. The success confirms the already verified validity of the employed computational methodology^8−10,28^ even when the molecular packing is strongly influenced by hydrogen bonding. In a more general way, these results indicate that the spectroscopic measurements, albeit limited to the Γ point, represent an extremely useful guideline to judge whether the calculations can in fact be employed to describe phenomena which depend on the complex dynamics of the different polymorphic structures.

The peculiarity of quinacridone and other pigments^11^ with respect to acene semiconductors is that the hydrogen bond adds to the interplay between van der Waals and π–π stacking in determining the crystal packing. It was indeed thought that the strong hydrogen bond network, inducing a tight face-to-face π–π arrangement, as opposite to the “detrimental” pentacene herringbone pattern, would favor charge transport. Band structure calculations^13^ and the transfer integrals reported in Table 7 indicate that this is not the case. In addition, the present results also indicate that the lattice distortion energy is comparable or is in any case an important fraction of the bandwidths. We have already remarked that for α^I^-QA transport is likely dominated by static and dynamic disorder, with a mechanism more similar to that of a semiconducting polymer. We believe that also in the case of the β-QA and γ-QA polymorphs, the observed appreciable mobilities^11^ cannot be easily accounted for by the current models of charge transport mechanism,^2^ where the possible influence of the hydrogen bond network is not considered. Additional work, like for instance time-of-flight mobility measurements and/or its temperature dependence, is required to confirm what is being suggested by the present results. In any case, they demonstrate the importance of the achieved reliable description of quinacridone lattice dynamics.

## Supporting Information Available

Crystal data of QA polymorphs, measurements in polarized light, Raman activities in polarized light for monoclinic structures, quinacridone gas-phase low-frequency normal modes, eigenvectors scheme of the β-QA normal modes at 116.3 and 122.6 cm^–1^, and frequencies of β-QA and γ-QA IR active phonons (PDF)
Animations for the lattice phonons modes provided by the calculations for QA polymorphs (AVI)
