# Supplementary material for: Lattice Dynamics of Quinacridone Polymorphs: A Combined Raman and Computational Approach
Source: Cryst Growth Des. 2023 Jul 31;23(9):6765–73. doi: 10.1021/acs.cgd.3c00634 (PMC10485816; doi:10.1021/acs.cgd.3c00634)
Supplement: Supplementary file 2 — cg3c00634_si_002.pdf [file cg3c00634_si_002.pdf]

Lattice dynamics of quinacridone polymorphs: a combined Raman and computational approach

Andrea Giunchi[‡ a] *Lorenzo Pandolfi*[‡ a,] *Raffaele G. Della Valle*[a], *Tommaso Salzillo*[*a], *Elisabetta Venuti*[*a], *Alberto Girlando*[*b]

[a]Dipartimento di Chimica Industriale "Toso Montanari", Università di Bologna, Viale del Risorgimento, 4, 40136, Bologna

[b]Molecular Materials Group, Strada Fontanini 68, 43124 Parma, Italy

Supporting information

TABLE OF CONTENT

Crystal data of QA polymorphs	S1
Measurements in polarized light	S2
Raman activities in polarized light for monoclinic structures	S3
Quinacridone gas phase low frequency normal modes	S4
Eigenvectors scheme of the β-QA normal modes at 116.3 and 122.6 cm^{-1}	S5
Frequencies of β-QA and γ-QA IR active phonons	S6

S1 Crystal data of QA polymorphs

Table 1: Crystallographic data of the quinacridone polymorphs α', β and γ [1]

Form	α'	β	γ
**Space group**	P1	$P2_1/c$	$P2_1/c$
**Z**	1	2	2
**a (Å)**	3.802(2)	5.692(1)	13.697(9)
**b (Å)**	6.612(3)	3.975(1)	3.881(3)
**c (Å)**	14.485(6)	30.02(4)	13.4020(10)
**α (deg)**	100.68(8)	90	90
**β (deg)**	94.40(6)	96.76(6)	100.44(1)
**γ (deg)**	102.11(5)	90	90
**Volume (Å^3)**	346.7(1)	674.5(9)	700.6(7)
**T (K)**	293(2)	293(2)	293(2)

S2 Spectra in polarized light

The simulation of the (unpolarized) spectra of a powder sample involves the calculation of the average over all possible rotations around the three Cartesian x,y,z axes of the laboratory reference frame and thus follows the same rules as gases, yielding for the scattered intensity I_{powder}:[26]

$$I_{powder} = \frac{45\alpha^2 + 7\beta^2}{45} \quad (1)$$

Where and α_{ij} are the elements of the polarizability matrix and α and β are the spherical and anisotropic part of the polarizability, respectively,

$$\alpha = \frac{\alpha_{xx} + \alpha_{yy} + \alpha_{zz}}{3} \quad (2)$$

$$\beta^2 = \frac{6\left(\alpha_{xy}^2 + \alpha_{xz}^2 + \alpha_{yy}^2\right) + (\alpha_{xx} - \alpha_{yy})^2 + (\alpha_{xx} - \alpha_{zz})^2 + (\alpha_{yy} - \alpha_{zz})^2}{2} \quad (3)$$

Similar to what happens in the experimental diffractogram of powders, however, the relative band intensities in the Raman measurements can be influenced by the occurrence of some predominant crystal faces in the sample.

When working with anisotropic samples such as crystals, it is necessary to consider how the sample is oriented in the measurement set up, in which the laboratory reference frame is defined. In the back scattering geometry used in this work the incident laser propagates in the *z*-direction and can be made polarized in the *x*- or *y*- directions, which lie on the measurement stage. Accordingly, the scattered light is analysed in the *z*-direction either parallel or perpendicular to the polarization direction of the incident laser light. Thus, there are four possible measurement combinations for a the crystal lying on the *xy* plane: i) incident and scattered polarizations both parallel to a selected crystal orientation; incident and scattered polarizations both perpendicular to it; iii,iv) incident and scattered polarizations perpendicular to each other in the two possible ways with respect to the crystal orientation. In Porto's notation[27] the combinations can be expressed as $\bar{Z}(XX)Z$, $\bar{Z}(YY)Z$, $\bar{Z}$ $(XY)Z$, $\bar{Z}(XY)Z$, where the first and last indexes indicate the direction of the propagation of the incident and scattered light, respectively, and the indexes in parenthesis their polarizations in the same order.

Once the experimental geometry is defined one needs to analyse how the elements of the polarizability tensor transform from the crystal reference frame into the laboratory reference frame. This corresponds to operate a basis change. If $\mathbb{R}$ is the transformation matrix from crystal to laboratory frame and $\mathbb{R}^T$ is its transpose, the polarizability tensor in the laboratory frame $\boldsymbol{\alpha}^{\boldsymbol{lab}}$ can be obtained by means of the similarity transformation $\mathbb{R}\,\boldsymbol{\alpha}^{\boldsymbol{crys}}\mathbb{R}^T$ of the crystal polarizability tensor $\boldsymbol{\alpha}^{\boldsymbol{crys}}$. Below we give the formulas of the Raman intensities deduced for each main crystallographic face of a monoclinic structure (such as that of polymorphs β- and γ- QA), for an arbitrary orientation of one of the crystal axes with respect to the polarization direction. The formulas for the same faces, assuming that one of the crystal axes is oriented with the light polarization are also given; as the light in the experimental setup is always propagating along the Z axis, this index can be neglected in all the expression.

S3 Raman activities and intensities in polarized light for monoclinic structures

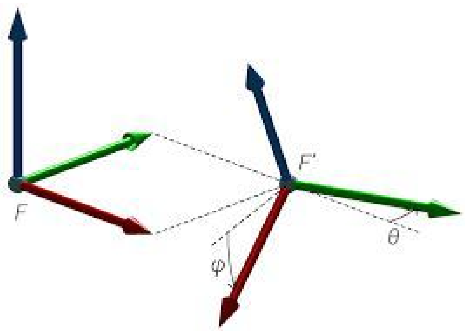

Arbitrary orientation of the crystal axes with respect to the polarization direction

$\underline{ab\ face}$

$$I_{\bar{Z}(XX)Z} = \left(a_{aa}\cos^2\varphi + \ a_{ab}\sin 2\varphi + \ a_{bb}\sin^2\varphi\right)^2 \qquad (4)$$
$$I_{\bar{Z}(XY)Z} = \frac{1}{4}(\,-a_{aa}\sin 2\varphi \ +2\ a_{ab}\cos 2\varphi + a_{bb}\sin 2\varphi)^2$$
$$I_{\bar{Z}(YY)Z} = \ \left(a_{aa}\sin^2\varphi - a_{ab}\sin 2\varphi + \ a_{bb}\cos^2\varphi\right)^2$$

$\underline{ac\ face}$

$$I_{\bar{Z}(XX)Z} = \left(a_{aa}\cos^2\varphi - \ a_{ac}\sin 2\varphi + \ a_{cc}\sin^2\varphi\right)^2$$
$$I_{\bar{Z}(XY)Z} = I_{\bar{Z}(YX)Z}\frac{1}{4}(a_{aa}\sin 2\varphi \ +2\ a_{ac}\cos 2\varphi - a_{cc}\sin 2\varphi)^2 \qquad (4)$$
$$I_{\bar{Z}(YY)Z} = \ \left(a_{aa}\sin^2\varphi + a_{ac}\sin 2\varphi + \ a_{cc}\cos^2\varphi\right)^2$$

$\underline{bc\ face}$

$$I_{\bar{Z}(YY)Z} = \{[a_{bb}\cos\varphi \ +(\ -a_{ab}\cos\beta + a_{bc}\sin\beta)\sin\varphi]\cos\varphi - \ [(a_{ab}\cos\beta - a_{bc}\sin\beta)\cos\varphi$$
$$I_{\bar{Z}(XY)Z} = \ \{[a_{bb}\sin\varphi \ +(\ a_{ab}\cos\beta - a_{bc}\sin\beta)\cos\varphi]\cos\varphi - \ [(a_{ab}\cos\beta - a_{bc}\sin\beta)\sin\varphi + \qquad (4)$$
$$I_{\bar{Z}(YY)Z} = \{[a_{bb}\cos\varphi \ +(\ -a_{ab}\cos\beta + a_{bc}\sin\beta)\sin\varphi]\cos\varphi - \ [(a_{ab}\cos\beta - a_{bc}\sin\beta)\cos\varphi$$

Samples oriented with one crystal axis parallel to the light polarization direction

$$I_{(XX)@ab} = \ \alpha_{aa}^2 \qquad (4)$$
$$I_{(XY)@ab} = I_{\bar{Z}(YX)Zab} = \alpha_{ab}^2$$
$$I_{(YY)@ab} = \alpha_{bb}^2$$

$$I_{(XX)@ac} = \ \alpha_{aa}^2 \qquad (5)$$
$$I_{(XY)@ac} = I_{\bar{Z}(YX)Z@ac} = \alpha_{ac}^2$$
$$I_{(YY)@ac} = \alpha_{cc}^2$$

$$I_{(XX)@bc} = \left[\left(\alpha_{aa}\cos^2\beta - \alpha_{ac}\sin 2\beta + \alpha_{cc}\sin^2\beta\right)\right]^2 \quad (6)$$

$$I_{(XY)@bc} = I_{\bar{Z}(YX)Z@100} = \left[\,\left(\alpha_{ab}\cos\beta - \alpha_{bc}\sin\beta\right)\right]^2$$

$$I_{(YY)@bc} = \alpha_{bb}^2$$

S4 Quinacridone gas phase low frequency normal modes

Standard Density Functional Theory (DFT) methods have been employed for the calculations of the harmonic normal modes of the quinacridone molecule in the gas phase using the Gaussian16 [2] quantum chemistry program, with the B3LYP/6-311++G** combination of functional and basis set. Frequencies were scaled by the 0.982 factor. [2]

Table S1 List of the harmonic vibrational frequencies of quinacridone in the gas phase in the frequency range 0-200 cm^{-1}

Frequency (cm^{-1})	Symmetry	IR intensity (Km/mol)	Raman activity Å^4/amu
29.21	A_u	2.04	0.00
59.04	A_u	5.37	0.00
64.74	B_g	0.00	930.14
110.46	B_u	5.10	0.00
123.80	A_u	1.84	0.00
133.86	B_g	0.00	81.97
185.85	A_u	1.02	0.00
188.43	B_g	0.00	27.55

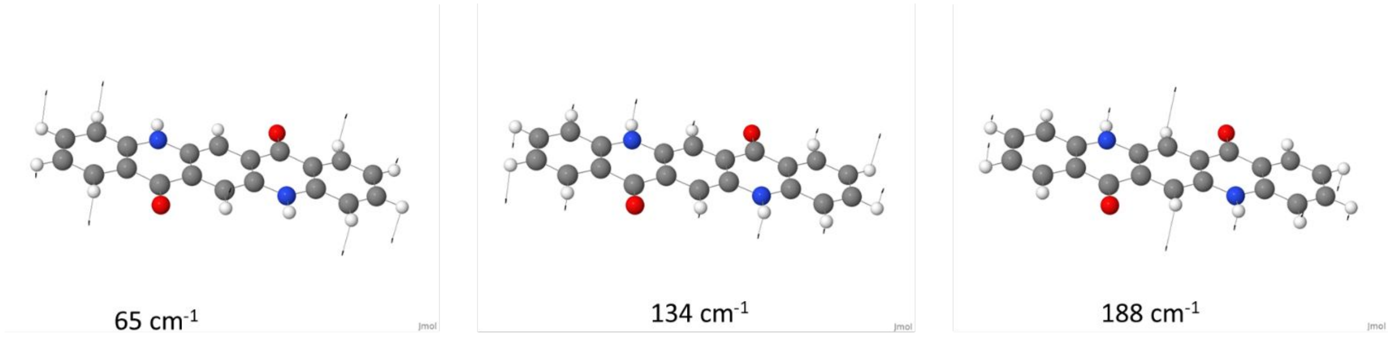

Figure S1 Scheme of the eigenvectors for the three quinacridone normal modes that are Raman active by symmetry and lie below 200 cm^{-1}

S5 Eigenvectors scheme of the β-QA normal modes at 116.3 and 122.6 cm^{-1}

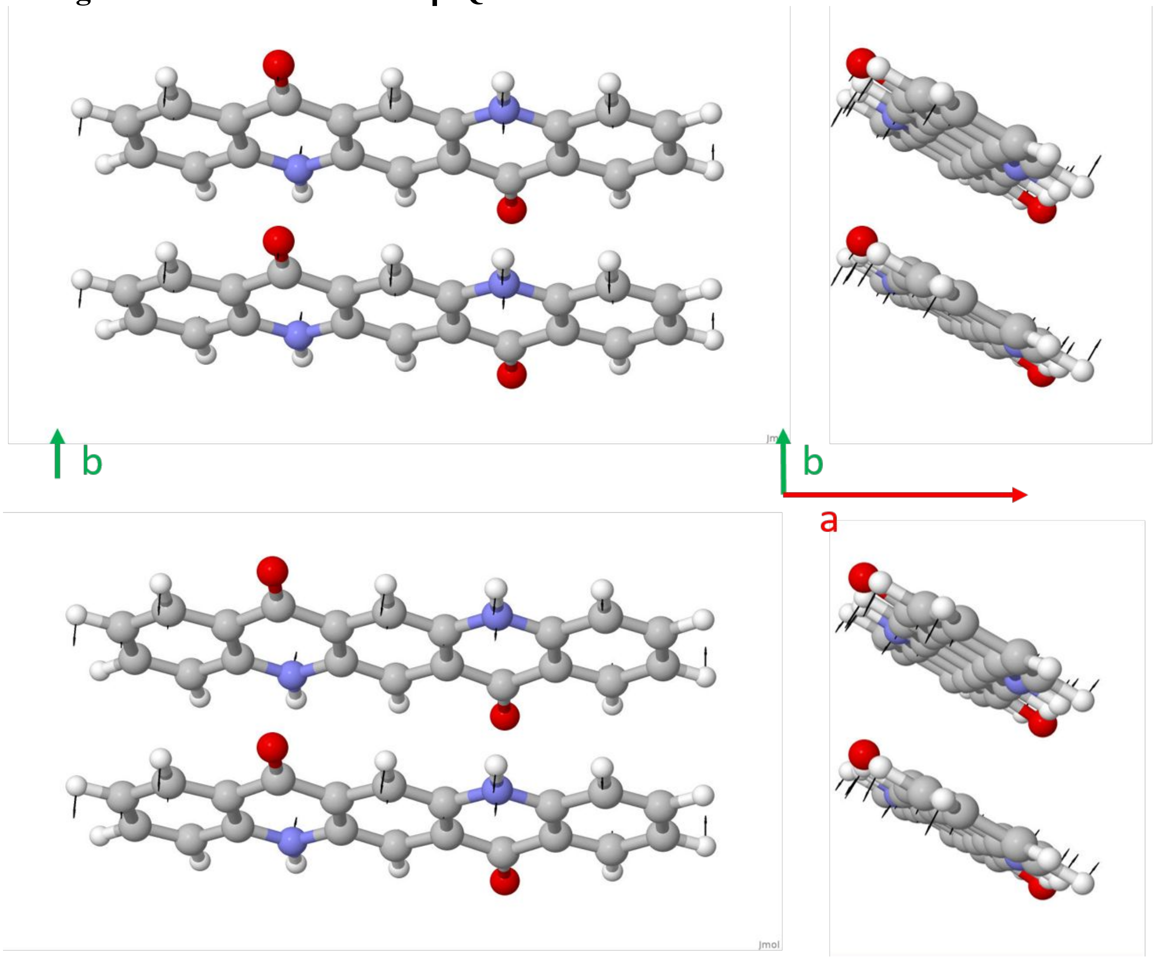

Figure S2 Scheme of the β-QA eigenvectors for the Raman active normal modes at 116.3 cm^{-1} (top) and 122.6 cm^{-1} (bottom) displaying the highest values of the Peierls coupling constants (around 11 meV) in the π π stacking direction (crystal b axis).

S6 Frequencies of β-QA and γ-QA IR active phonons

In the final stages of writing the present work, it came to our attention a recent paper reporting THz IR spectra and DFT calculations (CRYSTAL17 package, PBE functional, VTZ basis set with Grimme's D3 correction) of β-QA and γ-QA powders [3]. We did not calculate the IR intensities,

but of course we did obtain all the frequencies of IR active modes, that we compare in Table S2 with those reported in Ref. [3]. In general, there is good agreement between the two calculations, except for the lowest frequency phonons, that are missing both Ref. [3] experiment and calculation. But of course those phonons *must* be present, in correspondence with the Raman counterpart, and perhaps their experimental and computed very low intensity (see for instance their Fig. 7) have induced the authors to disregard them.

Table S2 Comparison of Ref. [3] experimental and computed frequencies (cm^{-1}) of β-QA and γ-QA with the results of the present calculation.

**β**-Quinacridone			***γ***-Quinacridone		
Ref. [3], Exp.	*Ref. [3], Calc.*	*This work, Calc.*	*Ref. [3], Exp.*	*Ref. [3], Calc.*	*This work, Calc.*
		18 B_u			47 B_u
		21 A_u			48 A_u
		32 A_u	64	61	60 A_u
		49 A_u	86	85	78 B_u
--	90	80 B_u	91	86	79 A_u
125	121	121 A_u	115	96	88 B_u
--	130	124 B_u	123	113	106 A_u
--	136	128 A_u	--	129	126 B_u
137	138	131 B_u	--	130	128 A_u
--	146	153 A_u	151	151	146 B_u
159	172	162 B_u	202	158	151 A_u
195	192	194 A_u	208	205	204 A_u
218	210	206 B_u	216	222	214 B_u

REFERENCES

1. E. F. Paulus, F. J. J. Leusen, and M. U. Schmidt, “Crystal structures of quinacridones,” *CrystEngComm*, vol. 9, no. 2, pp. 131–143, 2007.
2. Gaussian 16, Revision C.01, M. J. Frisch, G. W. Trucks, H. B. Schlegel, G. E. Scuseria, M. A. Robb, J. R. Cheeseman, G. Scalmani, V. Barone, G. A. Petersson, H. Nakatsuji, X. Li, M. Caricato, A. V. Marenich, J. Bloino, B. G. Janesko, R. Gomperts, B. Mennucci, H. P. Hratchian, J. V. Ortiz, A. F. Izmaylov, J. L. Sonnenberg, D. Williams-Young, F. Ding, F. Lipparini, F. Egidi, J. Goings, B. Peng, A. Petrone, T. Henderson, D. Ranasinghe, V. G. Zakrzewski, J. Gao, N. Rega, G. Zheng, W. Liang, M. Hada, M. Ehara, K. Toyota, R. Fukuda, J. Hasegawa, M. Ishida, T. Nakajima, Y. Honda, O. Kitao, H. Nakai, T. Vreven, K. Throssell, J. A. Montgomery, Jr., J. E. Peralta, F. Ogliaro, M. J. Bearpark, J. J. Heyd, E. N. Brothers, K. N. Kudin, V. N. Staroverov, T. A. Keith, R. Kobayashi, J. Normand, K. Raghavachari, A. P. Rendell, J. C. Burant, S. S. Iyengar, J. Tomasi, M. Cossi, J. M. Millam, M. Klene, C. Adamo, R. Cammi, J. W. Ochterski, R. L. Martin, K. Morokuma, O. Farkas, J. B. Foresman, and D. J. Fox, Gaussian, Inc., Wallingford CT, 2016.
3. M. P. Andersson and P. Uvdal. New scale factors for harmonic vibrational frequencies using the B3LYP density functional method with the triple-ζ basis set 6-311+G(d,p). The Journal of Physical Chemistry A, 109, 2937–2941, 2005.

4. A. D. Squires, A. J. Zaczek, R. A. Lewis, and T. M. Korter, Identifying and explaining vibrational modes of quinacridone *via* temperature-resolved terahertz spectroscopy: absorption experiments and solid-state density functional theory simulations. Phys. Chem. Chem. Phys. 22, 19672-19679, 2020.
